# Draft Genome Sequences of Five Putatively Novel *Saccharibacteria* Species Assembled from the Human Oral Metagenome

**DOI:** 10.1128/mra.00246-22

**Published:** 2022-06-27

**Authors:** Daniel Saito, Leandro Nascimento Lemos, Ana Tana Rosas Nascimento Ferreira, Cristiane Pereira Borges Saito, Rodrigo Ferreira de Oliveira, Fabiana de Souza Cannavan, Siu Mui Tsai

**Affiliations:** a Computational Biology Laboratory, Superior School of Health Sciences, Amazonas State University, Manaus, Amazonas, Brazil; b Functional Genomics Laboratory, Superior School of Health Sciences, Amazonas State University, Manaus, Amazonas, Brazil; c Cellular and Molecular Biology Laboratory, Center for Nuclear Energy in Agriculture, University of São Paulo, Piracicaba, São Paulo, Brazil; Indiana University, Bloomington

## Abstract

We report the draft metagenome-assembled genomes (MAGs) of five putatively novel *Saccharibacteria* strains retrieved from the oral microbiome. MAGs were obtained from nonstimulated saliva samples from hosts with various clinical statuses and correspond to distinct species taxonomically placed within the *Saccharimonadaceae* family, as determined by genome-wide analysis against previously described TM7 genomes.

## ANNOUNCEMENT

Saccharibacteria (TM7) are ultrasmall organisms that were originally detected in the open environment (1), some of which are common inhabitants of the oral microbiome associated with inflammatory conditions of the oral mucosal tissues (2). *Saccharibacteria* species harbor very peculiar genomes that might have coevolved as a reflection of their epibiontic lifestyle with other oral bacteria (3). In this study, the assembly of metagenome-assembled genomes (MAGs) retrieved from the oral metagenomes of subjects with different oral health statuses was performed, in an effort to further assess the breadth of *Saccharibacteria* diversity in the oral cavity.

Volunteers were recruited at the Dental Clinic of the Amazonas State University (Brazil) with no distinctions with respect to gender, race, or age. Written informed consent forms were signed by participants in accordance with the 7th version of the Declaration of Helsinki (2013). One-milliliter nonstimulated saliva samples were collected, subjected to DNA extraction with the MasterPure complete DNA and RNA purification kit (Epicentre) following the manufacturer's instructions, and quantified via a Qubit fluorometer (Thermo Fisher Scientific). Metagenomic DNA was mechanically fragmented with a proprietary hydrodynamic sonication protocol (Novogene HK), and 1.0 μg DNA was used for library preparation with the NEBNext Ultra DNA library preparation kit (New England Biolabs). Products in the range of 300 bp were selected and sequenced in a HiSeq 2500 instrument (Illumina) with a HiSeq SBS v.4 kit targeting 150-bp paired-end reads. Raw sequence quality control was performed with readfq_meta v.8 software (Novogene HK) by removing reads displaying more than 40 bp with Q values of ≤38 or presenting more than 10 undetermined (N) base pairs. Paired-end reads were merged and adapter sequences were removed with PEAR v.0.9.8. Human DNA was filtered out via genome mapping with Bowtie2 (4), and contig assembly was achieved with SPADES v.3.10.1 (5). MAGs were binned with Maxbin2, and completeness and contamination values were assessed with CheckM v.1.1.3 (6) according to minimum 50% completeness and maximum 10% contamination thresholds. Genome coverage was inferred with Bowtie2, and annotation was performed with both the KBase suite (7) and NCBI PGAP v.4.11. A search for antimicrobial resistance genes was conducted with CARDdb, and one for carbohydrate-active enzymes was performed with the dbCAN server. Taxonomic placement of MAGs was achieved with GTDB-Tk 1.7.0 (8) based on the Genome Taxonomy Database (GTDB) v.1.1.0 (9). An average nucleotide identity (ANI) value of 95% was adopted as the limit for species-level demarcation (10).

Of the 27 samples analyzed, only 5 yielded successful genome assemblies according to the adopted parameters, with clones OHS0006, OHS0013, and OHS0010 corresponding to healthy subjects and clones OPS0014 and OPS0017 to generalized chronic periodontitis cases. The reported genomes displayed <95% ANI values among themselves and with the 31 TM7 reference genomes available at GTDB, suggesting a direct correspondence to potentially novel *Saccharibacteria* species. General DNA sequencing statistics and annotation features of the announced MAGs are presented in Table 1.

**TABLE 1:** General information and annotation results for five MAGs belonging to the *Saccharimonadaceae* family, retrieved from the oral metagenome of distinct human individuals

Characteristic	Data for MAG:				
	OHS0006	OHS0010	OHS0013	OPS0014	OPS0017
BioProject accession no.	PRJNA717815	PRJNA717815	PRJNA717815	PRJNA717815	PRJNA717815
SRA accession no.	SRR14122728	SRR14122724	SRR14122746	SRR14122745	SRR14122742
BioSample accession no.	SAMN23242078	SAMN23242106	SAMN18522312	SAMN23242128	SAMN18718850
GenBank accession no.	JAKNRY000000000	JAKNSA000000000	JAGTWK000000000	JAKNSB000000000	JAGTWL000000000
Genus-level taxonomy	TM9x	Undefined	TM7x	UBA1105	Undefined
No. of reads	2,738,800	1,429,197	3,753,487	920,542	4,148,644
No. of contigs	175	217	39	248	21
*N*_50_ (bp)	4,877	2,333	44,498	3,222	96,171
Genome size (nt)	655,308	477,048	760,861	668,525	794,210
Genome coverage (×)	14.82	4.42	26.22	4.68	26.49
Completeness (%)	51.02	69.39	97.96	75.51	100.00
GC content (%)	43.40	41.30	43.15	51.21	47.36
Total no. of genes	816	618	834	778	836
No. of protein-encoding genes	765	572	780	731	787
No. of RNA genes	34	38	49	37	45
No. of tRNA genes	33	36	41	34	38
No. of noncoding RNA genes	1	1	2	1	4
No. of pseudogenes	17	8	5	10	4
No. of antibiotic target protection genes	5	4	4	7	5
No. of antibiotic target alteration genes	22	16	25	24	28
No. of antibiotic efflux genes	29	9	27	23	28
No. of antibiotic inactivation genes	1	1	0	1	2

### Data availability.

The final drafts of MAGs reported in this study, along with the respective raw sequence reads and BioProject and BioSample information, are publicly available at DDBJ/EMBL/GenBank under the accession numbers presented in Table 1.

## References

[1] Rheims H, Rainey FA, Stackebrandt E. 1996. A molecular approach to search for diversity among bacteria in the environment. J Indust Microbiol Biotechnol 17:159–169. doi:10.1007/BF01574689.

[2] Bor B, Bedree JK, Shi W, McLean JS, He X. 2019. Saccharibacteria (TM7) in the human oral microbiome. J Dent Res 98:500–509. doi:10.1177/0022034519831671. 30894042 PMC6481004

[3] Utter DR, He X, Cavanaugh CM, McLean JS, Bor B. 2020. The saccharibacterium TM7x elicits differential responses across its host range. ISME J 14:3054–3067. doi:10.1038/s41396-020-00736-6. 32839546 PMC7784981

[4] Langmead B, Trapnell C, Pop M, Salzberg SL. 2009. Ultrafast and memory-efficient alignment of short DNA sequences to the human genome. Genome Biol 10:R25. doi:10.1186/gb-2009-10-3-r25. 19261174 PMC2690996

[5] Bankevich A, Nurk S, Antipov D, Gurevich AA, Dvorkin M, Kulikov AS, Lesin VM, Nikolenko SI, Pham S, Prjibelski AD, Pyshkin AV, Sirotkin AV, Vyahhi N, Tesler G, Alekseyev MA, Pevzner PA. 2012. SPAdes: a new genome assembly algorithm and its applications to single-cell sequencing. J Comput Biol 19:455–477. doi:10.1089/cmb.2012.0021. 22506599 PMC3342519

[6] Parks DH, Imelfort M, Skennerton CT, Hugenholtz P, Tyson GW. 2015. CheckM: assessing the quality of microbial genomes recovered from isolates, single cells, and metagenomes. Genome Res 25:1043–1055. doi:10.1101/gr.186072.114. 25977477 PMC4484387

[7] Arkin AP, Cottingham RW, Henry CS, Harris NL, Stevens RL, Maslov S, Dehal P, Ware D, Perez F, Canon S, Sneddon MW, Henderson ML, Riehl WJ, Murphy-Olson D, Chan SY, Kamimura RT, Kumari S, Drake MM, Brettin TS, Glass EM, Chivian D, Gunter D, Weston DJ, Allen BH, Baumohl J, Best AA, Bowen B, Brenner SE, Bun CC, Chandonia J-M, Chia J-M, Colasanti R, Conrad N, Davis JJ, Davison BH, DeJongh M, Devoid S, Dietrich E, Dubchak I, Edirisinghe JN, Fang G, Faria JP, Frybarger PM, Gerlach W, Gerstein M, Greiner A, Gurtowski J, Haun HL, He F, Jain R, et al. 2018. KBase: the United States Department of Energy Systems Biology Knowledgebase. Nat Biotechnol 36:566–569. doi:10.1038/nbt.4163. 29979655 PMC6870991

[8] Chaumeil P-A, Mussig AJ, Hugenholtz P, Parks DH. 2019. GTDB-Tk: a toolkit to classify genomes with the Genome Taxonomy Database. Bioinformatics 36:1925–1927. doi:10.1093/bioinformatics/btz848. PMC7703759 31730192

[9] Rinke C, Chuvochina M, Mussig AJ, Chaumeil P-A, Davín AA, Waite DW, Whitman WB, Parks DH, Hugenholtz P. 2021. A standardized archaeal taxonomy for the Genome Taxonomy Database. Nat Microbiol 6:946–959. doi:10.1038/s41564-021-00918-8. 34155373

[10] Jain C, Rodriguez-R LM, Phillippy AM, Konstantinidis KT, Aluru S. 2018. High throughput ANI analysis of 90K prokaryotic genomes reveals clear species boundaries. Nat Commun 9:5114. doi:10.1038/s41467-018-07641-9. 30504855 PMC6269478

